# Applications of Technology to Record Locomotion Measurements in Dairy Cows: A Systematic Review

**DOI:** 10.3390/ani13061121

**Published:** 2023-03-22

**Authors:** Anna Bradtmueller, Amir Nejati, Elise Shepley, Elsa Vasseur

**Affiliations:** 1 Department of Animal Science, McGill University, Sainte-Anne-de-Bellevue, QC H9X 3V9, Canada; 2 Department of Veterinary Population Medicine, University of Minnesota, St. Paul, MN 55108, USA

**Keywords:** accelerometry, bovine, gait, kinematic, kinetic, locomotion

## Abstract

**Simple Summary:**

Lameness in cows is a major problem within the dairy industry that concerns both economics and animal welfare. The assigning of locomotion quality “scores” by human observers watching cows walk is a commonly used method for identifying lameness in dairy cows. However, automated gait assessment technologies have been developed as an alternative, and these can provide more detailed and objective data regarding locomotion. The three primary types of technology used for this purpose are kinetic, kinematic, and accelerometric, which involve looking at movement regarding space and time, forces associated with motion, and acceleration, respectively. We conducted a systematic literature review to determine measurements, and relationships between them, that have been recorded using these technologies, as well as through other methods. Within the 37 articles that were included, measurements recorded using these different technologies often overlapped. However, inconsistencies regarding details of the technologies and the approaches used made it difficult to compare specific locomotion measurements across studies. More research is needed to achieve a comprehensive understanding of how factors regarding the health, environment, and management of dairy cows affect locomotion, as recorded through the detailed, objective outcome measurements provided by these technologies.

**Abstract:**

Lameness within the dairy industry is a concern because of its associated costs and welfare implications. Visual locomotion scoring has been commonly used for assessing cows’ locomotion quality, but it can have low reliability and is relatively subjective compared to automated methods of assessing locomotion. Kinematic, kinetic, and accelerometric technologies can provide a greater number of more detailed outcome measurements than visual scoring. The objective of this systematic review was to determine outcome measurements, and the relationships between them, that have been recorded using kinematic, kinetic, and accelerometric technologies, as well as other approaches to evaluating cow locomotion. Following PRISMA guidelines, two databases were searched for studies published from January 2000 to June 2022. Thirty-seven articles were retained after undergoing a screening process involving a title and abstract evaluation, followed by a full-text assessment. Locomotion measurements recorded using these technologies often overlapped, but inconsistencies in the types of technology, the arrangement of equipment, the terminology, and the measurement-recording approaches made it difficult to compare locomotion measurements across studies. Additional research would contribute to a better understanding of how factors regarding the health, environment, and management of dairy cows affect aspects of locomotion, as recorded through the detailed, objective outcome measurements provided by these technologies.

## 1. Introduction

Methods of reliable gait assessment in dairy cows are of major interest for both producers and researchers. Abnormalities in gait can contribute to impaired locomotion or to lameness, which is “a deviation in gait resulting from pain or discomfort from hoof or leg injuries and disease” [1]. Lameness is a prevalent welfare concern and is considered the third most costly health problem in the dairy industry after mastitis and reduced fertility [2]. The early detection of lameness, or of gait abnormalities that may lead to lameness, can help minimize the costs and welfare concerns associated with impaired locomotion [3]. Producers and researchers have often relied on visual locomotion scoring systems as the primary method of gait assessment, as they are non-invasive, inexpensive, and relatively easy to carry out [4,5]. Visual locomotion scoring systems typically consist of a value given to represent the overall quality of gait on an analog scale (generally with a value from 0 to 100) or on a scale with multiple classes (commonly consisting of 3, 5, or 9 points), and has defined aspects and quality levels of gait for each score. Some visual scoring systems may also focus on specific attributes of gait, such as reluctance to bear weight on a limb or asymmetry of gait, which are also generally explained for observers via detailed charts. However, aspects of these visual scoring charts may be interpreted differently between individual observers, and inconsistencies between observers can lead to low inter- or intra-observer reliability [6]. The required training and time necessary to conduct locomotion scoring also make it less likely to be conducted frequently for on-farm purposes [7]. Therefore, lameness prevalence is often underestimated by producers [8]. Visual scoring may be conducted by an observer watching a recorded video to avoid the requirement of having a live observer physically present for long periods of time. One lameness scoring method was found to be generally comparable in levels of agreement between video and live scoring, with video scoring resulting in fewer false negatives of lameness [9]. However, recording video for the purpose of conducting gait or lameness scoring is not practical for on-farm purposes, and would not be an efficient alternative to live scoring for producers looking to assess gait within a herd.

To move beyond the limitations of visual locomotion scoring systems, several types of technology have been adopted to record measurements of locomotion at a more detailed level and through a more automated approach [7]. However, these technologies are often compared and validated against visual locomotion scoring methods, which are not an ideal reference point, as gait scoring is often prone to relatively more subjective interpretations of gait quality and low reliability within and between observers [6].

Technologies and methods that take a more indirect approach to assessing gait quality, or identifying changes in gait, through the recording of physiological and behavioral measurements that are associated with gait have also been used alongside visual gait scoring or other technological approaches of recording gait measurements. For example, infrared thermography has been used to record hoof temperature, which is a physiological measurement that may be associated with different levels of mobility due to the presence of hoof disorders affecting the temperature of the hoof [10]. Wearable sensors that are typically attached to a cow’s leg have been used to record behavioral measurements, such as activity and lying time, which may be affected by impaired locomotion [11]. These technologies and methods, which directly or indirectly evaluate gait, could provide alternative approaches to visual locomotion scoring for assessing locomotion from. Currently, however, there are gaps in the knowledge about what the differences within these technologies and methods are, as well as what specific measurements have been recorded using the different approaches.

Multiple literature reviews have been conducted that focus on technologies used for gait evaluation in dairy cows, although most of these primarily take a “lameness detection” approach. Two of these [3,12] focused only on wearable sensor technologies. One review of manual and automatic locomotion scoring systems for dairy cows was conducted with the aim of comparing and evaluating the agreement, reliability, and validity of manual and automatic locomotion scoring systems used in research [4]. This Schlageter-Tello et al. [4] review was also the first to highlight the issue of using visual locomotion scoring systems, which are more subjective compared to automatic systems and may have low reliability, as a reference for validating automated lameness detection systems. Another review [7] was conducted to describe the current automated systems—including kinematic, kinetic, and indirect methods—that are used for cattle lameness detection. The review, conducted by Alsaaod et al. [7], focused on the performance of the methods compared with a reference standard (locomotion score or lesion score), and described the technical aspects of these technologies, such as their levels of sensor technique, validation of their algorithms, their performance in lameness detection, and/or their decision support with an early warning system. Finally, a review conducted by Silva et al. [13], focusing on precision technologies used for addressing welfare concerns regarding dairy cattle, contained a section focusing on automated lameness detection, with technologies categorized as kinematic, kinetic, or indirect. While previous reviews focused on the technical aspects of technologies used in gait evaluation, our systematic literature review aims to focus on the specific measurements recorded by these different technologies and methods of assessing gait. Our review also aims to describe and draw out the relationships between specific types of measurement, both those that directly and indirectly assess cow locomotor ability. Our review will also lay out how individual studies define and go about recording specific types of measurement, as terms such as “step length” can often be measured with different approaches or have varying definitions between studies. Additionally, our review will be conducted from the perspective of analyzing gait in all aspects of locomotor ability, rather than from a lameness detection perspective. A sister scoping review by Nejati et al. [14] has also been conducted with the aims of mapping the research trends of quantitative bovine gait analysis, exploring the technologies that have been used to measure the biomechanics parameters of gait variables in bovine species, and highlighting the current gaps in the field of cow gait analysis. The sister review additionally covers trends in the frequency of use that the three technologies of primary interest (kinematic, kinetic, and accelerometric) within the current study have had in research since the year 2000. These aspects will, therefore, not be covered here.

The objectives of this systematic literature review consisted of two parts. Firstly, we wanted to determine what specific measurements have been taken using our three technology types of primary interest—kinetic, kinematic, and accelerometric—to directly measure dairy cow locomotor ability. Secondly, we wanted to determine what other approaches outside of these three technologies have been used to record locomotion measurements. In looking outside these three technologies, we additionally wanted to consider the physiological and behavioral measurements that have been used in other approaches of gait assessment to indirectly evaluate cow locomotor ability. After determining what measurements were being recorded to evaluate locomotion and how they were recorded, we aimed to map the relationships between these different types of direct measurement of gait and indirect, gait-related measurements.

## 2. Materials and Methods

This review was conducted using guidelines adapted from the Preferred Reporting Items for Systematic Reviews and Meta-Analyses [15].

### 2.1. Eligibility Criteria

The eligibility criteria required that studies include 2 of the 3 following levels to be considered appropriate for addressing the objective of this review. These characteristics, along with their definitions, are as follows:

A. Level A: the use of one or more of the three autonomous technologies of primary interest (kinematic, kinetic, and accelerometric) for directly evaluating gait through the recording of locomotion measurements.
B. Level B: the use of approaches that evaluate gait through the recording of:
  I. Locomotion measurements recorded through other methods (human observer-based) or technologies (less autonomous technologies outside of kinetics, kinematics, and accelerometry).
  II. Gait-associated physiological (e.g., hoof temperature, mechanical nociception threshold, and muscle fatigue) or behavioral measurements (e.g., activity and lying time).
C. Level C: the presence of a relevant study context (environmental or cow-level risk factor(s) that measurements recorded through the Level A and B approaches are used to evaluate in research).

Note regarding studies appearing to meet a combination of B and C: For the purposes of study selection for this review, visual locomotion scoring conducted by humans was considered a Level B approach to assessing gait; however, studies that use only visual locomotion scoring to assess gait quality/locomotor ability when evaluating factors that may affect locomotion were not included.

Primary research in the English language was included, and review papers and conference proceedings were excluded so that only studies with original peer-reviewed research relevant for addressing the objective of this review would be used. Validation and technology, algorithm, and model development studies were excluded, as studies focusing only on the technical aspects (validity, sensitivity, and specificity) of the technologies discussed were not relevant to the objective of the review. Studies using certain measurements, evaluation methods, or technologies that, in some cases, could be deemed as locomotion-related, but that focused on topics other than locomotion (GPS/animal tracking, estrus detection, calving detection, behavior monitoring independent of locomotion, etc.), were also excluded due to a lack of relevance. Only papers from the year 2000 and after were included because the technology and methods used before that time would be outdated and no longer of use or relevance in the current research. Studies were also required to use adult dairy cows as subjects, as this review focuses only on locomotion in adult dairy cows, and species, animal production type, stage of life, and sex can influence the locomotion of an animal. 

### 2.2. Information Sources and Search Strategy

Literature searches were conducted in two electronic databases (Scopus and Web of Science Core Collection) to obtain references. The final search was conducted on 1 June 2022. The search terms were designed to include all relevant keywords and to ensure results with the greatest number of possible references. Four levels of search terms were developed. In the final search, only the first three levels of keywords were incorporated into the search queries, and the fourth level was excluded to ensure that the maximum number of relevant references resulted from the search terms. The query strings that were used in each database and the number of records that resulted from each are shown in Table 1. The “Combination 1, 2, and 3” rows contain the queries and search results that were ultimately used for the reference screening process to ensure that the maximum number of potentially relevant articles underwent the screening process. No search limitations were set regarding language, date, study subject, or study design to minimize bias and ensure that all relevant references could be obtained.

Additional searches were later conducted to obtain any relevant references that had not been included in the initial database searches. Supplementary searches included forward- and back-searches of references obtained from the initial database searches, as well as hand searching to gather individual references missed by the database searches.

### 2.3. Study Selection

**Figure 1:**
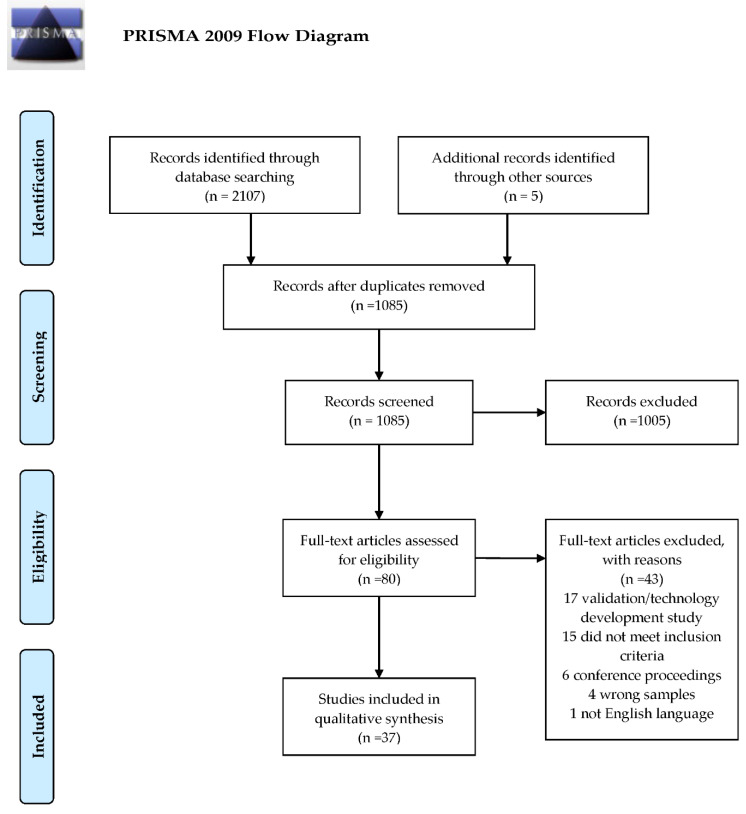
PRISMA flow diagram for systematic review detailing the selection and screening of literature.

All resulting references from the “Combination 1, 2, and 3” strings were imported into Endnote X8 reference management software. Duplicates were then removed, and the remaining references were screened using the web application Rayyan (Rayyan, Qatar Computing Research Institute). A two-step screening process was used. The first step consisted of the screening of reference titles and abstracts to determine relevance to the review objectives and research questions, as well as other general eligibility criteria, such as language and date requirements. References incorporating at least two of the three A, B, and C eligibility levels listed above were then included in the second step of screening. The second step consisted of a full-text review to confirm that the references met the eligibility criteria. The study selection process is shown in Figure 1.

### 2.4. Data Collection Process, Data Items, and Summary Measurements

Data extraction sheets were developed by the authors to chart the literature. Screening and data extraction were conducted by an individual reviewer. Specific definitions for the technology categories of kinematics, kinetics, and accelerometry, which are provided in the results section, were determined by the reviewers before the data extraction process. Uncertainties regarding the review process or protocol were discussed with the review team to minimize human error. The initial data extraction sheet consisted of the headings: reference, direct measurement(s) of locomotor ability, technology category for direct measurement(s) (kinetic, kinematic, accelerometer), indirect measure(s) associated with locomotor ability, factor investigated (cow-level or external factor), methodology, country where the study was conducted, non-locomotion-related measurements, recording interval/duration, number of animals, number of farms, treatment/comparisons, difference(s) between treatments/comparisons, *p*-value, conclusions, and limitation(s)/critique(s). Additional charts corresponding to more specific aspects within the review objective were later developed to organize the data further.

**Figure 2:**
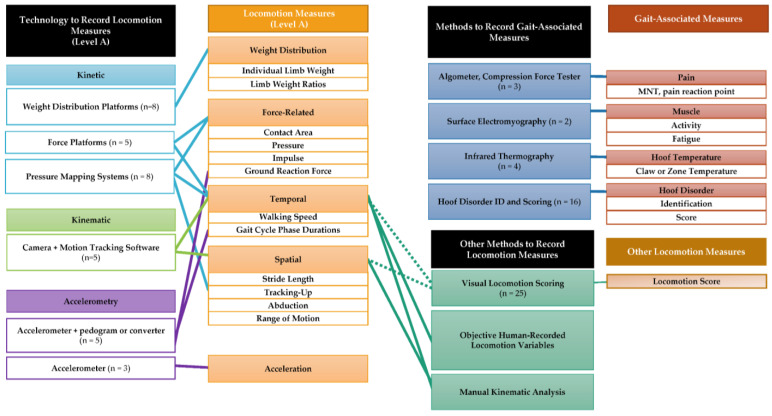
Relationship diagram of types of measurement recorded in the studies in this literature review and the methods used to record them. (Note: Not all measurements are listed for each measurement category. Examples of common measurements are displayed. Additionally, types of behavioral, gait-associated measurement and the approaches to recording them are not included, but are explained in the “Approaches to recording physiological and behavioral gait-associated measurements” section).

A narrative synthesis and the organization of tables based on the research questions of the review were used to summarize and present the data. Tables and/or narrative descriptions were developed for each objective and its subsequent research questions. Definitions and categorization terms were developed by the review team to ensure consistent and thorough descriptions of the measurements used. Relevant data items were organized based on the types of technology and the methods used for gait analysis. A visual diagram (Figure 2 in results Section 3.7) was developed to display the connections and relationships between the types of measurement recorded using different technologies and methods of gait assessment.

## 3. Results and Discussion

### 3.1. Study Selection

A total of 1342 references in Scopus and 765 references in Web of Science resulted from the combination of search strings 1, 2, and 3. After deduplication was conducted, 1085 references remained for title and abstract screening. An additional five references were found from hand searching. Based on a lack of relevance to bovine locomotion and obvious discordance with the eligibility criteria, 1005 papers were excluded. Eighty references remained for full-text screening. Through full-text examination, 43 references were excluded, leaving a final number of 37 references. Figure 1 shows a PRSIMA flow diagram for the selection and screening process of the review.

### 3.2. Approaches to Recording and Analyzing Gait in Dairy Cows: Categorizations and Definitions

In research that evaluates factors that may impact dairy cow locomotion, the three main categories of technologies used to record direct measurements of locomotion are kinematic, kinetic, and accelerometric [14]. These types of technology are generally more autonomous and provide more detailed measurements of locomotion than other types of technology that have been used for similar purposes. Therefore, in this review, these three technologies are of primary interest, as are the locomotion measurements that they record. Measurements recorded using one type of technology, but whose definitions may be associated with another type of technology, will be organized and described under the section pertaining to the technology through which they were recorded.

Other methods outside of these three technology types have also been used to record measurements of locomotor ability. In this review, gait or gait-associated measurements recorded via technologies or other methods that kinetics, kinematics, and accelerometers are categorized as “Level B”, with sub-categories detailing how they approach recording locomotion or gait-associated measurements. For example, Level B encompasses methods such as “manual kinematics”, which involve the recording of kinematic-type measurements through software that is not specifically designed for gait analysis but has been used with the goal of recording locomotion measurements. The use of image analysis software or custom-written code generally requires a greater amount of manual human work to obtain kinematic measurements than the gait analysis-specific software encompassed within Level A, which allows for easier, automated “tracking” of movement. Additionally, even simpler approaches, or “human-recorded locomotion variables”, to recording kinematic-type measurements have been used. Human observations—live or via video recordings—have been conducted to record easily identifiable measurements such as the number of steps taken within a passage. Stopwatches or timers have been used for recording the time taken for a cow to walk a known distance to allow for a simple approach to calculating walking speed. Finally, methods involving human observations for looking at overall locomotor ability or specific gait characteristics, such as scores from numeric rating scales or analog locomotion rating scales, have been commonly used.

### 3.3. Kinematics

Kinematics is a subdivision of the study of biomechanics that involves observable aspects of motion, such as space and time [16]; thus, measurements recorded using kinematic technology generally fall under the categories of “spatial” and “temporal.” Spatial measurements provide information as to how the body is moving within space, and temporal measurements provide information as to how the body is moving in time. The type of technology that has mainly been used to record kinematic measurements in research that evaluates factors affecting dairy cattle locomotion is a combination of video cameras alongside commercially available motion analysis software. These video and software systems, which are designed specifically for motion analysis, are the sole technology to be considered as “kinematic” technology in this review. However, other types of technology that are designed to record kinetic measurements have also been used to record kinematic measurements. In this review, these kinetics-focused technologies and all the measurements that they record will be discussed under the “kinetics” section. Additionally, five studies included in this review used accelerometers in conjunction with a specific pedogram or converter designed to extract certain spatial or temporal measurements. While spatial and temporal measurements would generally fall under the category of kinematics, these five studies will be discussed under the “accelerometry” section of the review. 

The Level A type of kinematic technology used for recording spatial and temporal measurements in these studies involved the use of commercially available motion tracking or motion image analysis software specifically designed for the analysis of kinematics. These studies used these technologies with only a single camera to record video, which means that only one side and one angle of the cow was visible, and thus, the cow’s gait was recorded such that in some cases, kinematic measurements were recorded for only two ipsilateral limbs. Cameras were often placed at different distances from the “walkways”, which also had varying dimensions between studies. Three different software programs were used within these studies, and they all required markers to be attached to the cow. This type of system allows for 2D kinematic analysis, where the marker movement is “tracked” to provide data, which then can be extracted and interpreted as specific spatial and/or temporal measurements or variables. The use of this technology allows for the recording of detailed, quantitative gait measurements, such as stride time, which can then be used to compare between cows or between an individual cow’s gait cycles, to assess gait change over time, or to assess contralateral limb movements. While commonly used, visual locomotion scoring may identify when a cow experiences a deviation from its “normal” gait, or may be able to pinpoint a deviation from what would be “normally” expected regarding a specific gait characteristic, these more detailed and quantitative measurements can go a step further and provide data representing motion trajectories of specific points on the cow’s body, through which patterns can be detected and a potential cause of gait impairment can be inferred. Additionally, recording multiple interconnected spatial and temporal measurements for multiple body parts at once can provide a clearer picture of a cow’s locomotion overall as well as what is occurring at different phases within the gait cycle.

#### Measurements Recorded by Kinematic Technology 

Spatial measurements recorded using kinematic technologies in dairy cow locomotion research generally look at the distance between two points—either between two points on the cow or between the floor and a point on the cow—or at the range of movement of a particular part of the body. Studies using the above video and motion analysis software systems to evaluate dairy cow locomotion have used spatial measurements of stride length, tracking distance, the length of particular regions of the spine, the range of movement for different joints, and head position, and measurements that describe the maximum height a particular point on the cow reaches during locomotion (specific definitions for each variable are detailed in Table 2). All five studies measured stride length, three measured hoof height, and three measured tracking. For these measurements, the definitions and approaches used to record them were similar. Only one study recorded the spatial posture measurements of head position, spine markers of height, spine length, thoracic region length, and lumbar region length, with each being recorded during a frame of video when the cow was seen to be bearing weight on the same/a consistent limb throughout. These measurements were recorded to provide information regarding the posture of the cow. Blackie et al. [11] and Blackie et al. [17] both recorded hock rotation of movement (ROM) and fetlock ROM. Blackie et al. [11] was the only study that recorded the ROM of the knee.

Temporal measurements that were recorded in these studies include durations of specific parts of the gait cycle. Three studies measured stride duration, stance duration, and swing duration. The definitions and approaches used for these measurements were relatively similar (Table 2). Only one study recorded triple support. The walking speed of the cow can also be calculated based on recorded kinematic variables. Three studies using automatic motion tracking software—as opposed to the Level B kinematic approaches of “manual kinematics” or “human-recorded kinematics”—recorded walking speed, although two of these did so by calculating the walking speed based on the stride duration divided by the stride time, and the third did not provide a definition as to how walking speed was calculated (Table 2).

Within the studies using direct kinematic technology approaches to recording kinematic measurements, definitions between studies remained fairly consistent. However, for studies using only manual kinematic approaches to record kinematic measurements, differences between how different types of software used work and how images or videos are processed between studies could result in greater inconsistencies in what could otherwise appear to be similar types of measurement.

### 3.4. Kinetics

Kinetics is a subdivision in the study of biomechanics that focuses on the forces associated with motion [16]. In research evaluating dairy cattle locomotion, technologies using kinetic measurements can be divided into three general categories: force platforms (FPs), pressure mapping systems (PMSs), and weight distribution platforms (WDP) [14]. Force platforms and pressure mapping systems may be used independently or in a system where the two are combined to record simultaneously. All three of these types of kinetics-focused technologies may record “static” measurements, or measurements taken as the cow is standing in place over the platform. However, force platforms and pressure mapping systems are generally used to record “dynamic” measurements, or measurements taken as the cow walks over the platform. While kinetic technologies primarily focus on measuring kinetic (force-related) measurements, FPs and PMSs may also record kinematic-type spatial or temporal measurements. Kinematic-type measurements that have been recorded using kinetic technologies in studies evaluating dairy cow locomotion will be discussed in this section. Details of the measurements recorded using these three technologies are provided in Table 3.

**Table 3:** Locomotion measurements recorded and analyzed using force platforms, pressure mapping systems, and weight distribution platforms.

Technology and Measurements	Measurement Description/Approach	References
**Force Platform ^1^**		
**Force-Related ^2^**		
Ground reaction force (GRF)	Average ground reaction force of a tested limb normalized by the animal’s dynamic weight	[21]
	Vertical GRF exerted to the lateral and medial claw (parameters analyzed for five moments (heel strike, maximum braking, midstance, maximum propulsion, and push off) of stance phase for the left and right limbs)	[22]
	Vertical (Fv), longitudinal (Fl), and mediolateral (Fm) ground reaction forces	[23]
Maximum/peak force	Maximum force per lateral and medial claw (used for analysis deceleration, midstance, and acceleration positions)	[24]
	Maximum force per foot (used for analysis deceleration, midstance, and acceleration positions)	[24]
	Peak GRF of a tested limb normalized by the animal’s dynamic weight	[21]
	Symmetry index for peak GRF (a pelvic limb symmetry variable)	[21]
	Positive cranio-caudal peak force	[25]
	Negative cranio-caudal peak force	[25]
	Vertical peak 1 (fore and hind limbs)	[25]
	Vertical peak 2 (hind limb parameter only)	[25]
	Vertical peak 3 (hind limb parameter only)	[25]
Force asymmetry	Symmetry index for average GRF (a pelvic limb symmetry variable)	[21]
	Symmetry parameters calculated for vertical (Fv), longitudinal (Fl), and mediolateral (Fm) ground reaction forces to compare entire stance phase curves of the left and right legs; 0 to 100 scale signifying stance phase curve symmetry (lower values signifying better symmetry/more parallel left and right leg curves)	[23]
GRFω	Area under the Fourier transformed curve of a GRF signature normalized by the animal’s dynamic weight	[21]
	Symmetry index GRFω (pelvic limb symmetry variable)	[21]
Impulse	The integral of the GRF normalized by the animal’s dynamic weight with respect to time	[21]
	Symmetry index for vertical impulse (a pelvic limb symmetry variable)	[21]
	Positive cranio-caudal impulse	[25]
	Decelerative impulse	[25]
	Accelerative impulse	[25]
Moment of force (torque)	Moment of vertical (Fv), longitudinal (Fl), and mediolateral (Fm) ground reaction forces	[23]
**Kinematic: Temporal ^2^**		
Stance time	Period of time a limb is in contact with the floor	[21]
Stance time asymmetry	Symmetry index for stance time (a pelvic limb symmetry variable)	[21]
Zero crossing	% stance	[25]
Stride frequency	Not provided	[25]
Swing time	Not provided	[25]
Walking speed	Calculated from the GRF data using timing information (frame number) and COP co-ordinates corresponding to forelimb mid-stance on two different force plates; speed is (in m/s) divided by distance between COP location in stride one, and stride two, by the difference in time, calculated from the difference in frame numbers divided by the sample rate (200 Hz)	[25]
Duty factor	Not provided	[25]
**Pressure Mapping System ^1^**		
**Force-Related ^2^**		
Force	Force for left/lame foot and right/non-lame foot (to compare)	[26]
	Force for each foot (4) (basic gait, within-imprint variable)	[27]
	Static vGRF (while cow is standing)	[28]
	Dynamic vGRF (while cow is walking)	[29]
	Total vertical force during locomotion (relative value as a percentage of hoof strike average)	[30]
	Corrected mean vertical force claw–floor interactions during locomotion (per footprint double-support phase time average)	[30]
	Shapes of force–time curves assessed for local maxima and where they were in the stance phase	[29]
Force asymmetry	Symmetry in force between left and right limbs	[27,31]
Maximum force	Maximum vertical force per sensor	[30]
Impulse	Impulse for both the lame/left and non-lame/right feet (to compare)	[26]
Weight distribution	Calculated using at least eight pairs of vertical impulses from steady state locomotion recordings of each cow; hind limb vertical impulse is then expressed as a percentage of forelimb impulse for each plate; mean of all the ratios is calculated to determine mean weight distribution across all cows	[25]
Contact area	Dynamic overall loaded area (while cow is walking)	[29]
	Claw–floor contact area during locomotion (relative value as a percent of hoof strike average)	[30]
	Mean claw–floor contact area during locomotion (per footprint double-support phase time average)	[30]
	A_zone_: the loaded area per zone relative to the total zone area	[28]
	Static total loaded area/overall contact area (while cow is standing)	[28,29]
Pressure	Static mean pressure (while cow is standing)	[28,29]
	Dynamic mean pressure (while cow is walking)	[29]
	Pav: average pressure per foot at five moments of stance phase (heel strike, maximum braking, midstance, maximum propulsion, and push off)	[22]
	Contact pressure for both the left/lame and right/non-lame feet (to compare)	[26]
	COPx: center of pressure in a lateromedial direction	[30]
	COPy: center of pressure in a craniocaudal direction	[30]
	vGRF per loaded area per zone (Pzone) (describes the pressure in each zone)	[28]
Maximum pressure	Static maximum pressure (while cow is standing)	[28,29]
	Dynamic maximum pressure (while cow is walking)	[29]
	Pmax: maximum pressure per foot at five moments of stance phase (heel strike, maximum braking, midstance, maximum propulsion, and push off)	[22]
**Kinematic: Spatial ^2^**		
Stride length	Not described	[26]
	Not described	[31]
	Distance between two consecutive imprints of the same hoof	[27]
Step length asymmetry	Step length symmetry between left and right limbs	[27]
Tracking	Step overlap or tracking up	[31]
	The lengthwise distance between the front hoof imprint and a subsequent imprint of the hind hoof on the same side	[27]
Abduction	The sideways distance between the front hoof imprint and a subsequent imprint of the hind hoof on the same side	[27]
Step width asymmetry	Step width symmetry between left and right limbs	[31]
	Mean difference in step width between left and right hoof imprints	[27]
Distance between hoof imprints	Ax: relates to the distance between hoof imprints along the X dimension	[31]
	A_Y_: relates to the distance between hoof imprints along the Y dimension	[31]
	A_T_: relates to the distance between hoof imprints along the t dimension	[31]
Distance within hoofprints	B_X_: relates to the distance within hoof imprints along the X dimension	[31]
	B_Y_: relates to the distance within hoof imprints along the Y dimension	[31]
	B_T_: relates to the distance within hoof imprints along the t dimension	[31]
**Kinematic: Spatio-Temporal ^2^**		
Transversal deviations for each foot	Relative location and timing of imprints in X direction (between-imprint variable)	[27]
Coefficients of variation of transversal deviations for each foot	Represent stride-to-stride fluctuation of transversal deviations for each foot (an inconsistent gait variable)	[27]
Longitudinal deviations for each foot	Relative location and timing of imprints in Y direction (between-imprint variable)	[27]
Coefficients of variation of longitudinal deviations for each foot	Represent stride-to-stride fluctuation of longitudinal deviations for each foot (an inconsistent gait variable)	[27]
Step time (T)	Relative location and timing of imprints in the T direction/dimension	[27]
**Kinematic: Temporal ^2^**		
Stance time	Time during one stride that the hoof is on the floor	[31]
	Stance time symmetry between left and right limbs	[27]
Stance time asymmetry	Stance time symmetry between left and right limbs	[31]
	Not described	[27]
Stride time	Not described	[27,31]
Step time	Not described	[31]
	Step time symmetry between left and right limbs	[27]
Step time asymmetry	Not described	[31]
**Weight Distribution Platforms ^1^**		
**Weight Distribution^2^**		
Limb weight ratio	Ratio of weight placed on legs (maximum weight asymmetry)	[32]
	Ratio of weight on hind legs	[33,34]
Mean limb difference	Δweight(%): mean weight difference across the healthy and the lame limb within the affected limb pair	[33,35,36,37]
Limb weight	Mean weight applied to each limb	[33,35,36]
	Mean percentage of weight applied to each limb	[38]
	Mean percentage of weight distributed on front pair and back pairs of legs	[38]
Mean variation	Mean variation of weight distributed on each limb	[38]
Standard deviation of weight applied to limb	A measure to determine weight shifting between hind limbs	[33,35,36]
Mean standard deviation of weight applied	Mean SD of weight applied to all 4 legs	[32]
	Mean SD of weight applied to rear legs and mean SD of weight applied to front legs	[34]

^1^ Technology type. ^2^ Measurement category.

#### 3.4.1. Force Platforms

Five studies included in this review used FP technology for dairy cow locomotion analysis. The recording of force-related measurements may provide insight into the cow’s gait by primarily focusing on differences in force applied between legs as the cow steps. The presence of hoof disorders or injuries on a particular leg make it likely that the cow will load less weight on that hoof, and therefore, will step down on the hoof with relatively less force than the hoof of her contralateral limb. Determining the differences between the forcefulness of the steps on contralateral limbs is also a method for evaluating gait symmetry. Three of the studies used force plates independently to record force-related measurements. Force plates used alone were supported by load cells, placed in pits to be level with the ground of the surrounding walkway, and covered with rubber mats to provide additional friction to the walking surface. Liu et al. [21] and Thorup et al. [23] had FP systems arranged so that two force plates were parallel and could record both sides of the cow’s body, with dimensions allowing for two to four stances to be recorded on each plate. Walker et al. [25], alternatively, created a 3 m long, 0.9 m wide walkway using five smaller force plates arranged in a row, with the goal of collecting data from a pair of limbs on one side of the body.

Two studies used FP technology in conjunction with pressure mapping systems. Carvalho et al. [24] mounted a PMS on top of a force platform consisting of a metal base plate with load cells at the corners supporting a top metal plate. The PMS and force platform both had the same dimensions so that the FP could measure the correct force under any individual limb, and then, that force could be used to calibrate the PMS. Van Der Tol et al. [22] used a Kistler force plate placed underneath a PMS. They sampled simultaneously so that the force plate could output a vertical ground reaction force that could be used for calibration of the PMS. The total force measured by the FP was also used to adjust the sum of vertical forces that were applied to the individual sensors of the PMS.

##### Measurements Recorded by Force Platforms

Studies using FP technology primarily recorded measurements of GRF, which are the vertical or three-dimensional ground reaction forces applied to the surface of the platform, and measurements related to GRF, such as the pressure and moment of force. Details regarding the definitions of each of these measurements and the approach used to record them are shown in Table 3. However, these measurements are often organized as a more specific type of variable, usually consisting of a calculation involving multiple sub-measurements, to investigate the aspect of locomotion that is of interest. Studies using FP technologies independently (without a PMS) were only used to record dynamic measurements, which were primarily organized into variables or scales focusing on gait symmetry. Liu et al. [21] presented measurements recorded using the StepMetrix system as “limb movement variables”, and reported the force-related measurements of the peak ground reaction force, average ground reaction force, vertical impulse, and GRFω. Thorup et al. [23] measured vertical, longitudinal, and mediolateral ground reaction forces, as well as their associated moments (torque). Both studies used force measurements to evaluate gait symmetry. Liu et al. [21] developed a symmetry index for average GRF to evaluate pelvic limb symmetry, while Thorup et al. [23] developed symmetry parameters calculated for GRFs in each dimension to compare entire stance phase curves between the left and right legs. Thorup et al. [23] used a scale from 0 to 100 to represent stance phase curve symmetry, with lower values signifying more parallel left and right leg curves, and thus, better symmetry. Walker et al. [25] recorded several types of peak in GRF curves, as well as three types of impulse measurement. Weight distribution was also calculated from a minimum of eight pairs of vertical impulses from steady state locomotion recordings of each cow. The two studies using FPs in conjunction with PMSs [22,24] primarily relied on PMSs to record kinetic measurements, with the FP used as an accessory technology. The kinetic measurements recorded in these studies will be described in the following PMS section. Two studies looking at temporal aspects of gait used force plates to record kinematic-type measurements. Liu et al. [21] measured stance time and developed a symmetry index for stance time to evaluate pelvic limb symmetry. Walker et al. [25] measured stride time, stride frequency, swing time, walking speed, and zero crossing. 

#### 3.4.2. Pressure Mapping Systems

Eight studies included in the review used pressure mapping systems (PMSs). Pressure mapping systems are unique as a kinetic technology, as they are the only technology to have a network of sensors, allowing for the identification of multiple hoofprints of different limbs during one passage, as opposed to FPs, which can only record the sum of force occurring on one platform/sensor. This allows PMSs to record a broader range of both kinematic and kinetic measurements. Carvalho et al. [24] and Kleinhenz et al. [26] used the Matscan pressure measuring system (Tekscan Inc., South Boston, MA, USA. Oehme et al. [28,29] used the Hoof™ System (M3200E, Tekscan Inc., Boston, MA, USA), a foil-based piezoresistive pressure measurement system. It is important to note the difference between the two studies in which the Hoof™ System was used, as Oehme et al. [28] used amputated hooves attached to a load applicator to press down on the film, while Oehme et al. [29] cut the pressure film to be in the shape of claw and fitted the insoles into leather claw shoes that were attached to the cow. Van Nuffel et al. [27] was the only study included in the review to use the GAITWISE system, which was developed by Maertens et al. [39] and has a greater length (6 m); this allows for data to be recorded for up to three consecutive gait cycles. Van Nuffel et al. [31] used a permanently installed pressure distribution plate, which was a precursor to the later-developed GAITWISE system. Van Der Tol et al. [22] and Ouweltjes et al. [30] both used Footscan pressure distribution plates (RsScan International, Olen, Belgium); however, Van Der Tol et al. [22] used the pressure distribution plate on top of a Kistler force plate (Kistler Corp, Winterthur, Switzerland), while Ouweltjes et al. [30] used the Footscan 2D-box system (RsScan International, Olen, Belgium), which was used independently of a force plate. Carvalho et al. [24] also used a force plate underneath the Matscan system. 

##### Measurements Recorded Using Pressure Mapping Systems 

Pressure mapping systems used in conjunction with force plates may record force through their associated force plates, while PMSs used independently can extrapolate force based on the pressure and contact area measured. Five of the seven studies included in the review that used PMSs recorded measurements of force, although different approaches were used across studies. Oehme et al. [29] recorded both “static” and “dynamic” force, while Oehme et al. [28] recorded only a static measurement of force, as it was an ex vivo study using an amputated hoof attached to a load-applicator. Van Nuffel et al. [27,31] both recorded variables looking at asymmetry of force. Five studies using PMSs recorded some type of measurement of pressure, and four recorded contact area. Kleinhenz et al. [26] was the only study to use PMSs to record impulse.

In addition to recording force-related measurements, PMSs are also used to record spatial and temporal measurements of gait. Although PMSs are a kinetic-type technology, the timing and distance of hoofprints upon the platform can be used to calculate kinematic measurements. Spatial measurements that have been measured using a PMS include stride length, tracking up, abduction, and asymmetry variables relating to spatial measurements. Van Nuffel et al. [31] also recorded measurements of distance within hoofprints and distance between hoofprints in different spatial dimensions. Temporal measurements that have been recorded using a PMS include stride time, stance time, and step time. Van Nuffel et al. [27,31] also recorded stance time symmetry between left and right limbs. These kinematic-type measurements often used the same terminology as studies using kinematic visual motion-tracking approaches to recording kinematic measurements, although clear definitions and calculations used to obtain these measurements were not always provided. Further details on measurements recorded through a PMS are shown in Table 3.

#### 3.4.3. Weight Distribution Platforms

Weight distribution platforms are technologies that measure weight distribution to evaluate aspects of locomotion, especially with regard to lameness detection. WDP technologies are more frequently being utilized within milking robots as automated milking systems (AMS) grow in popularity, although none of the studies included in this review involve a WDP within an AMS. Compared to other kinetic technologies, WDPs are more limited in the types of measurement they can provide, as they only record “static” measurements—measurements taken while the cow stands—of weight distribution across limbs. They may measure weight distribution within one instant, or across a short period of time, to evaluate the shifting of weight between limbs. Thus, they provide an objective alternative to the subjective, visual observation of a cow’s reluctance to bear weight on a particular limb, which is a method commonly used when an overall gait score or the specific gait characteristic of limping is considered. 

Two types of WDP technology have been used in research evaluating factors that may influence dairy cattle locomotion. The first is an Itin + Hotch weighing platform (Futterungstechnik, Liestal, Switzerland) consisting of four independent recording units with one hermitically sealed load cell (HBM, Volketswil, Switzerland) each, which has been used in five studies [33,35,36,38,40]. The second is a Pacific Industrial Scale weighing platform (Richmond, British Columbia, Canada) consisting of four independent recording units each containing four hermetically sealed load cells (Anyload LLC, Santa Rosa, CA, USA, which has been used in two studies [32,41].

##### Measurements Recorded using Weight Distribution Platforms

Studies using WDPs to evaluate aspects of dairy cattle locomotion have recorded several different types of specific measurement relating to weight distribution among cows’ legs. The limb weight ratio among either all four legs or between only the hind legs has been used as a measurement of maximum weight asymmetry in several studies. The mean limb difference, which describes the weight difference across a healthy and a lame limb within a pair of limbs, has also been recorded. Other measurements used include the mean weight applied to each limb and the standard deviation of weight applied to individual limbs, which allows for the determination of weight shifting between hind limbs. Finally, the mean standard deviation of weight applied to multiple limbs—either to all four, to the rear legs, or to the front legs—has also been recorded. Details of the measurements recorded are shown in Table 3.

For all studies using kinetic technologies, differences in factors such as the thickness of the rubber mats placed over platforms, the recording frequency, and the filters or adjustments made to raw recordings should be taken into account. Across the six studies using WDP, two brands of commercially available WDP were used. While recording frequency, which was sometimes varied, all these studies had the goal of measuring weight distribution amongst limbs, and therefore, calculated ratios which could be more easily compared across studies than those measurements recorded in studies using FP or PMS technologies.

### 3.5. Accelerometry

Accelerometers are used in biomechanics for the purpose of recording acceleration. While accelerometry is a kinematic-related technology, the purpose of accelerometers is to primarily measure acceleration. Acceleration as a measure can be compared between limbs to identify an impaired limb or an abnormality in gait. Other kinematic-type variables can also be extrapolated from the recorded acceleration. For the purposes of this review, accelerometers are considered their own category of gait-assessment technology, as they are used differently and, generally, with greater ease and fewer limitations than kinematic technologies or PMSs used to record kinematic-type measurements. Accelerometers that have been used to evaluate aspects of animal behavior, rather than to evaluate locomotion specifically, will be discussed in the gait-associated measurements section.

One type of accelerometer that has been used to record acceleration for the purpose of cow locomotion analysis is the Hobo Pendant G Acceleration Data Logger (Onset Computer Corp., Bourne, MA, USA). Chapinal et al. [34] used five of these accelerometers, with four attached to the lateral side of each leg above the fetlock and one attached to the right of the dorsal midline. Franco-Gendron et al. [20] used two of these accelerometers, which were each attached to a rear leg above the fetlock. One study, which aimed to measure the acceleration of the whole cow rather than of individual legs, used the acceleration sensing system Vibration Measurement Pack MVP-A3 (MicroStone, Nagano, Japan). The sensor was placed at the posterior end of the thoracic vertebrae of the cow to measure vertical, forward, and lateral acceleration. Specific software (Vibration Measurement Pack1.7.5, MicroStone) was used to manage the system, and the storage device for the sensor was attached to the collar of the cow. Other studies have used accelerometers in conjunction with a validated “converter” or pedogram designed to extract kinematic and kinetic gait cycle variables from the acceleration data, rather than focusing on acceleration as the outcome measure itself. Alsaaod et al. [42] used a 3D accelerometer, the Rumiwatch (ITIN + HOCH GmbH, Fütterungstechnik, Liestal, Switzerland), attached to the proximal side of a rear leg fetlock joint, along with RumiWatch Manager 2 software (Version 2.1.0.0, ITIN + HOCH GmbH, Liestal, Switzerland) to extract kinematic variables from the acceleration data. Alsaaod et al. [37] validated a system, the Cow-Gait-Analyzer pedogram, which extracts kinematic and kinetic gait cycle variables from the output of a USB Accelerometer X16-4 (GulfCoast Data Concept, Waveland, MS, USA). Multiple studies have used this combination of technologies to assess gait [35,36,40,43].

#### Measurements Recorded using Accelerometric Technology

In studies using accelerometers to measure acceleration directly, the measurements used were the mean acceleration and the asymmetry of variance of acceleration, which was meant to represent the irregularity of stepping patterns for the rear limbs [20,34]. Tanida et al. [44] measured the mean acceleration and variance of acceleration separately for the vertical, lateral, and forward directions. In one study using accelerometers, along with a validated “converter”, the kinematic outcomes obtained using RumiWatch Manager 2 were walking time, walking bouts, stride number, stride frequency, stride duration, and stride distance [42]. For studies using accelerometers in conjunction with the Cow-Gait-Analyzer pedogram, the kinematic outcomes were gait cycle duration, relative stance phase duration, and relative swing phase duration. The kinetic outcomes were foot load and toe-off, which are the maximum acceleration of the initial ground contact of the claw and of the termination of the ground contact of the tip of the claw, respectively [43]. In three studies, these measurements, extracted from the Gait-Analyzer pedogram, were used alongside measurements of weight distribution recorded via WDP to evaluate how gait changed after lameness intervention surgery [35], after lameness treatment [40], and after the administration of an analgesic to alleviate pain from lameness [36]. The combination of these technologies allowed for both static and dynamic kinetic measurements of locomotion, along with the kinematic outcome measurements of the pedogram, providing multiple approaches to recognizing how lameness was specifically impacted in these intervention studies. However, while these extrapolated kinematic and kinetic outcome measurements would be comparable between studies, they could not be directly compared to measurements from studies that recorded kinematic- and kinetic-type measurements through kinematic-focused or kinetic-focused technologies. Details regarding locomotion measurements recorded via accelerometers are shown in Table 4.

### 3.6. Other Approaches to Recording Locomotion Measurements

“Manual kinematic” approaches often involve the use of software outside of that focused on gait analysis, such as software that allows for the processing of images from a recorded video of a cow walking. For example, Tanida et al. [44] calculated the range of vertical and forward movement in each limb by looking at the difference in the maximum and the minimum value of pixels on the x- and y-axes. Manual kinematic analysis may also involve the use of various types of software to calculate the walking speed of a cow within a video by recording the time taken for the cow to walk between two points of a known distance [25,41]. Additionally, variables such as walking speed, step speed, and the number of strides per passage have also been recorded by humans via live observations with stopwatches or observations as cows pass between two physical markers in video recordings. Several studies used these “manual kinematic” or objective “human-observed locomotion variable” approaches, along with measurements of locomotion recorded using Level A kinematic, kinetic, or accelerometer technologies [20,25,45]. In some cases, these measurements were used as a way to validate measurements being recorded via Level A technologies, while in other cases, they were used as an additional method of recording a locomotion measurement that was not recorded via other technologies.

Visual locomotion scoring was a frequently used method of providing an overall score for gait or for providing scores of individual characteristics or attributes of gait, such as joint flexion. Twenty-five studies in this review used locomotion scoring (also termed gait, mobility, or lameness scoring) to assess the locomotor ability of cows, with several more studies using locomotion score in the process of selecting animals to be included in studies. Multiple types of numeric rating system (most commonly, 3- or 5-point scales), and analog scales (typically marked on a line representing values from 0 to 100) were used. Additionally, defining gait characteristics that were scored or used to determine an overall gait score varied between scoring approaches.

### 3.7. Approaches to Recording Physiological and Behavioral Gait-Associated Measurements

Multiple approaches to recording measurements have been used that do not directly focus on gait characteristics, but rather, may be associated with changes in locomotor ability or factors that may contribute to impaired locomotor ability. Sensors, accelerometers, and live observations have been used for recording measurements, which may help gain insight into a cow’s locomotor ability, such as measurements providing information relating to how the cow is distributing her time (e.g., lying time vs. time spent active; 12), as well as other physiological or other direct or indirect behavioral indicators of lameness (e.g., rumination). Although these behavioral measurements are often recorded via accelerometer technology, they are considered “gait-associated” measurements within this review, as they are not direct measurements of locomotion. Approaches using surface electromyography (SEMG) [45,46], infrared thermography (IRT) [11,26,47,48] or surface temperature data loggers [46], hoof pain measurement devices [21,26,49], ultrasonography [47], and hematology [26,33,47,50,51] have been used for recording physiological measurements. Additionally, hoof disorder identification and scoring methods have been commonly used to subjectively record the presence and severity of various hoof pathologies. These “gait-associated” measurements have been used in addition to locomotion measurements recorded through Level A technologies, or independently of locomotion measurements, for the purpose of comparing or exploring relationships between multiple types of potentially gait-associated measurements. Most of these studies have thus worked to combine gait-associated variables with locomotion scoring to investigate the relationships between these variables. The categorizations and relationships of gait-focused and gait-associated measurements and the technologies and methods used to record them can be found in Figure 2. Finally, hoof and leg injuries or disorders that often cause locomotor impairment may be associated with other types of measurement relating to the general health of the cow. Studies in this review that recorded general health measurements generally focused on those related to stress or immunology, such as heart rate or immunological gene expression.

## 4. Limitations, Implications, and Future Research

The results of this review indicate that a wide variety of approaches have been used to record similar types of gait measurement across multiple technologies and procedures. For instance, multiple visual locomotion scoring methodologies were used, and although most of the studies primarily used the same two or three scoring protocols and referenced them, the use of different protocols could create variation in how researchers interpret and consider specific aspects of cow locomotion when designing studies or planning how to utilize technologies for locomotion assessment. Locomotion scores, as a comparison, provide a very general idea of a cow’s gait and lack the ability to provide an actual objective value for a more specific type of measurement. For example, many numeric rating scales used for locomotion may focus on “asymmetry” as an aspect of gait that is evaluated by an observer. However, when we look at studies using direct technologies, many of them look at differences between more specific types of measurement, such as stride duration, stride length, or acceleration between contralateral limbs, as an approach to measuring asymmetry. Moreover, different scoring systems included or prioritized different sub-variables of gait, and potentially had greater influence on researchers who followed one protocol to focus on a particular aspect of gait than on another researcher who followed a different protocol when considering the overall gait quality of a cow. For example, the commonly used Flower and Weary [52] locomotion scoring system instructs observers to evaluate and provide scores for the sub-variable of head bob, whereas the commonly used Sprecher et al. [53] scoring system does not. While scoring protocols are referenced, details on how to score a specific sub-variable are often not reported; hence, sub-variables could be interpreted differently between observers conducting scoring. Therefore, more specifications pertaining to a scoring methodology should be reported, along with a reference to the scoring system used, creating homogeneity amongst information reported and leading to a more objective comparison of results and utilization of protocols. If one were to go a step further to conduct meta-analyses with data from these types of studies, several factors, including (i) differences in the technologies used, (ii) the approaches taken, (iii) equipment arrangements, (iv) technical aspects of the equipment used, and (v) the terminology of outcome measurements, would need to be taken into consideration.

This review excluded studies that were solely focused on the validation of a technology or on developing algorithms or models utilizing locomotion assessment technology. While the objective of this exclusion was to allow for a focus specifically on locomotion measurements being recorded in studies that investigated specific factors that could affect cow gait, these types of validation or model development studies are also important in this area of research. As locomotion assessment technologies become increasingly applied to dairy cows both in research and commercially, opportunities to use data with machine learning approaches to investigate locomotor issues further will continue to arise. In studies aimed at developing and validating new technologies or models, it is again recommended that any human-observer locomotion scoring methodology used be reported with as much detail as possible to ensure transparency and allow for evaluations and comparisons of different technologies used across studies. As these technologies advance and their potential for on-farm commercial application grows, considerations regarding their ease of use, cost-effectiveness, and efficiency at detecting locomotor abnormalities early on in a management setting should be researched further. In a scoping review conducted by Nejati et al. [14] looking at research trends in quantitative bovine gait analysis technologies, pressure mapping systems were deemed to be the most practical on-farm kinetic technology (including pressure mapping systems, force platforms, and weight distribution platforms) for assessing cow gait quality. This was due to their greater ease of implementation and ability to provide more diverse outcome measurements regarding gait. Computer vision, which uses deep learning technology, and accelerometers that can be attached to the cow are expected to be the next emerging areas of research in bovine quantitative gait analysis technologies [14]. It is expected that more “hands-off” approaches of automated gait assessment will continue to be researched since they are more likely to have success with on-farm commercial use. These technologies would eventually need to be optimized to create alerts for producers, and considerations regarding the severity level of a gait abnormality for which an alert is generated, and the frequency of alerts, would need to be made. Bioeconomic modeling could be useful for gaining insight into how practical a locomotion assessment technology might be for on-farm implementation within a certain area and/or management system.

A future implication of the continued adoption of these technologies in research could be that more data will become available that provides insight into the extent to which specific aspects of a cow’s environment or management—such as housing type, flooring surface, bedding, or hoof trimming practices—impacts the development or exacerbation of mobility issues. As these technologies are used in research in place of traditional human-observer locomotion scoring methods, results from new studies may differ from or contradict those of previous studies using human scoring since these technologies would be providing greater amounts of and more reliable quantitative and objective data. Once systems utilizing these technologies are optimized for on-farm application and begin replacing the gait assessment of cows that has been performed through producer observation alone or the use of older technologies to indirectly detect lameness, such as accelerometers recording activity and lying time, improved reporting of true lameness prevalence on farms could occur. This may result in a higher lameness prevalence being reported in general, as it is suspected that current reports underestimate its true prevalence [5]. However, the increased possibility of earlier detection and intervention of gait abnormalities with the on-farm application of locomotion assessment technology could result in the reported number of severe clinical lameness cases decreasing over time.

## 5. Conclusions

The use of automated locomotion or lameness assessment technologies is of great interest to both producers and researchers. The accurate and early detection of signs of locomotor impairment that would not require the training, time commitment, and sometimes, low reliability of visual locomotion scoring would be beneficial for on-farm gait evaluation. Kinematic, kinetic, and accelerometric technologies are alternative approaches that can evaluate specific aspects of locomotion with a greater level of detail and provide a greater number of outcome measurements than visual locomotion scoring. However, the inconsistencies between studies in how these technologies are set up to record locomotion measurements demonstrates that kinematic, kinetic, and accelerometric technologies are still in relatively early stages of use in dairy cow locomotion research and, therefore, on-farm application. The multiple other methods for evaluating locomotion that have been used to circumvent the limitations of these technologies, such as the recording of physiological or behavioral measurements associated with gait or more manual methods of recording locomotion measurements, also encompass a wide range of measurement types and approaches that are difficult to compare across studies. These other approaches also do not provide the straightforward, detailed locomotion measurements that are possible to obtain through kinematics, kinetics, and accelerometry. Additional research using these three technologies, as well as technical advancements and the development of strategies to overcome their current limitations, are needed to fully evaluate how various factors regarding the health, environment, and management of dairy cows may specifically change dairy cow movement. Further knowledge gained from future research applying these technologies will help to enhance their on-farm application for routine use.

## Figures and Tables

**Table 1:** The strings included in the search strategy with the number of resulting records for each string in the Scopus database on 1 June 2022. The “Combination 1, 2, and 3” rows contain the queries and search results that were ultimately used for the reference screening process.

Number	String	Records Found
1	TITLE-ABS (cattle OR cow * OR bovine)	703,838
2	TITLE-ABS (locomot * OR movement OR gait OR walk *)	1,818,651
3	TITLE-ABS (kinemat * OR kinetic * OR thermography OR electromyography OR * emg OR hematology OR sensor * OR ams OR “Automatic milking system” OR “milking robot” OR accelerometer *)	4,382,560
4	TITLE-ABS (exercise OR “outdoor access” OR pasture OR flooring OR “hoof health” OR “leg health” OR lameness OR environment * OR risk * OR hous *)	11,133,385
1, 2, 3	TITLE-ABS (cattle OR cow * OR bovine) AND (locomot * OR movement OR gait OR walk *) AND (kinemat * OR kinetic * OR thermography OR electromyography OR * emg OR hematology OR sensor * OR ams OR “Automatic milking system” OR “milking robot” OR accelerometer *)	1346
1, 2, 3, 4	TITLE-ABS (cattle OR cow * OR bovine) AND (locomot * OR movement OR gait OR walk *) AND (kinemat * OR kinetic * OR thermography OR electromyography OR * emg OR hematology OR sensor * OR ams OR “Automatic milking system” OR “milking robot” OR accelerometer *) AND (exercise OR “outdoor access” OR pasture OR flooring OR “hoof health” OR “leg health” OR lameness OR environment * OR risk * OR hous *)	418

* Qualifier to illustrate all possible endings of a word, i.e., Excit* could be excitement, excitation, exciting.

**Table 2:** Locomotion measurements recorded and analyzed using a camera and kinematic motion analysis software.

Measurements	Measurement Definition/Approach	References
**Spatial: Limb Movement ^1^**		
Stride length	Horizontal displacement between 2 consecutive hoof strikes of the same hoof	[18,19]
	Distance between 2 consecutive hoof strikes for the same hoof (right side of cow only)	[20]
	Distance between cannon appearing straight and next occurrence of cannon being straight for the fore and hind limbs	[11,17]
Hoof height	Maximum vertical displacement between 2 consecutive hoof strikes of the same hoof	[18,19]
	Maximum vertical distance at which the hoof is lifted while the cow is walking	[20]
Maximum fetlock height	Highest distance from the floor to the fetlock marker that weas seen during the stride	[17]
Maximum hock height	Highest distance from the floor to the hock marker that was seen during the stride	[17]
Tracking	Horizontal distance between front hoof strike and subsequent ipsilateral rear hoof strike	[18]
	Distance between the fore foot being placed on the ground and the ipsilateral hind foot being placed on the ground	[11,17]
Hock range of motion (ROM)	Difference between minimum and maximum hock angles, calculated by tracking the hind fetlock, hock, and stifle markers	[11,17]
Fetlock ROM	Difference between minimum and maximum fetlock angles, calculated by tracking the fetlock marker, knee marker, and elbow marker	[11,17]
Knee ROM	Difference between minimum and maximum knee angles, calculated by tracking the fore fetlock marker, knee marker and elbow marker	[11]
**Spatial: Posture ^1^**		
Head position	Distance from bottom of cow’s nose to floor (measured when front right foot is first observed to bear weight)	[17]
Spine markers height	Distance from the spine markers to the floor (assessed when cow is seen to be bearing weight on front right foot)	[17]
Spine length	Distance between markers at T3 and TA (assessed when cow is seen to be bearing weight on front right foot)	[17]
Thoracic region length	Distance between markers at T3 and L1 (assessed when cow is seen to be bearing weight on front right foot)	[17]
Lumbar region length	Distance between markers at L1 and SA (assessed when cow is seen to be bearing weight on front right foot)	[17]
**Temporal: Individual Limb ^1^**		
Stride duration	Time interval between 2 consecutive hoof strikes of the same hoof	[18,19]
	Not described	[17]
Stance duration	Period of time when the hoof is in contact with the ground (interval between hoof strike and following hoof-off) (right side of cow only)	[18,19]
	Period of time when a cow’s hoof is on the ground during a stride	[20]
Swing duration	Period of time when the hoof is not in contact with the ground (interval between toe-off and following hoof strike)	[18]
	Period of time when a cow’s hoof is on the ground during a stride (right side of cow only)	[20]
**Temporal: Overall ^1^**		
Walking speed	Stride length ÷ stride duration	[18,19]
	Not described	[11]
Triple support	Time spent with 3 hooves in contact with the ground; calculated as (sum of intervals between toe-off and subsequent contralateral hoof strike ÷ stride duration) × 100	[18]

^1^ Measurement category.

**Table 4:** Locomotion measurements recorded and analyzed using accelerometers independently and in conjunction with a validated pedogram.

Technology and Measurements	Measurement Description/Approach	References
**Accelerometer^1^**		
**Acceleration ^2^**		
Acceleration	Mean acceleration	[20,34]
	Forward lateral and vertical acceleration (to describe pattern of acceleration of whole body of cow)	[44]
Acceleration variance	Mean variance in acceleration of cows’ backs before and after hoof trimming	[44]
Acceleration asymmetry	Asymmetry of acceleration variance (%)	[20,34]
**Accelerometer + cow** **Cow-Gait-Analyzer pedogram ^1^**		
**Kinematic ^2^**		
Gait cycle duration	Interval between 2 consecutive foot load peaks	[43]
Stance phase duration	Percentage of time claw is in contact with the ground relative to the total gait cycle	[35,36,40,43]
Swing phase duration	Percentage of time in swing phase relative to total gait cycle	[35,36]
**Kinetic ^2^**		
Foot load	Maximum acceleration of the initial ground contact of the claw	[35,36,40,43]
Toe-off	Maximum acceleration of the termination of the ground contact of the tip of the claw	[35,36,40,43]

^1^ Technology type. ^2^ Measurement category.

## Data Availability

The data presented in this study are available within this article.
